# The Effect of Chronic Psychological Stress on Lower Urinary Tract Function: An Animal Model Perspective

**DOI:** 10.3389/fphys.2022.818993

**Published:** 2022-03-21

**Authors:** Yunliang Gao, Larissa V. Rodríguez

**Affiliations:** ^1^Department of Urology, The Second Xiangya Hospital of Central South University, Changsha, China; ^2^Department of Urology, Keck School of Medicine, University of Southern California, Los Angeles, CA, United States

**Keywords:** chronic psychological stress, lower urinary tract dysfunction, bladder, animal model, mechanism, treatment

## Abstract

Chronic psychological stress can affect urinary function and exacerbate lower urinary tract (LUT) dysfunction (LUTD), particularly in patients with overactive bladder (OAB) or interstitial cystitis–bladder pain syndrome (IC/BPS). An increasing amount of evidence has highlighted the close relationship between chronic stress and LUTD, while the exact mechanisms underlying it remain unknown. The application of stress-related animal models has provided powerful tools to explore the effect of chronic stress on LUT function. We systematically reviewed recent findings and identified stress-related animal models. Among them, the most widely used was water avoidance stress (WAS), followed by social stress, early life stress (ELS), repeated variable stress (RVS), chronic variable stress (CVS), intermittent restraint stress (IRS), and others. Different types of chronic stress condition the induction of relatively distinguished changes at multiple levels of the micturition pathway. The voiding phenotypes, underlying mechanisms, and possible treatments of stress-induced LUTD were discussed together. The advantages and disadvantages of each stress-related animal model were also summarized to determine the better choice. Through the present review, we hope to expand the current knowledge of the pathophysiological basis of stress-induced LUTD and inspire robust therapies with better outcomes.

## Introduction

Stress is an adaptive reaction of the organism in response to the effects of different external and internal adverse events (or stressors) (Chrousos, 2009). Psychological stress in humans can be regarded as a chain of events that disrupt homeostasis due to the direct effect of stress on the mind (Schneiderman et al., 2005). Short-term psychological stress can act as a strong motivator to perform better. However, chronic psychological stress may lead to maladaptive adjustments in homeostasis, which includes pathological effects on metabolism, vascular function, nerve system, and others (Wingfield and Sapolsky, 2003). In particular, chronic psychological stress has been perceived clinically as a potential risk factor that affects urinary function and exacerbates the symptoms of patients with lower urinary tract (LUT) dysfunction (LUTD), most profoundly in overactive bladder (OAB) and interstitial cystitis–bladder pain syndrome (IC/BPS) (Macaulay et al., 1987; Lutgendorf et al., 2001; Rothrock et al., 2001; McVary et al., 2005; Fan et al., 2008; Zhang et al., 2013; Bradley et al., 2014; Lai et al., 2015a,^2016^). For example, multiple studies have found that adults with LUTDs display a positive relation to affective disorders such as anxiety and depression (Rothrock et al., 2001; Coyne et al., 2009). Similarly, children with LUTDs commonly report psychological symptoms and disorders (Oliver et al., 2013). The causative effects of psychological stress on LUTD can be further supported by the findings from veterans, who with psychiatric comorbidities were significantly more likely to have a LUTD diagnosis (Klausner et al., 2009; Bradley et al., 2014, 2017; Breyer et al., 2014). Therefore, chronic psychological stress may play a role in both the development and exacerbation of bladder symptoms.

Despite the findings from clinical studies, the pathophysiological mechanisms of chronic psychological stress-induced LUTD have still not been clearly defined. Instead of the inherent limitations (e.g., legal, ethical, and moral limitations) of clinical studies, the application of stress-related animal models facilitates further exploration and acknowledgement of the interplay of psychological stress and LUTD. These animal models appear to share multiple key characteristics of LUTDs such as increased voiding frequency (Smith et al., 2011), enhanced bladder pain (Lee et al., 2015), or bladder distension (Chang et al., 2009). Currently, a substantial number of animal models have been developed to study stress-induced LUTD, and the commonly used models include water avoidance stress (WAS) (Cetinel et al., 2005; Saglam et al., 2006; Robbins et al., 2007; Zeybek et al., 2007; Okasha and Bayomy, 2010; Smith et al., 2011; McGonagle et al., 2012; Yamamoto et al., 2012; Bazi et al., 2013; Lee et al., 2015; Ackerman et al., 2016; Gao et al., 2017; Matos et al., 2017; Dias et al., 2019; Kullmann et al., 2019; Holschneider et al., 2020a,b; Sanford et al., 2020; West et al., 2021), social stress (Chang et al., 2009; Wood et al., 2009, 2012, 2013; Mann et al., 2015; Mingin et al., 2015; Weiss et al., 2015; Wang et al., 2017; Butler et al., 2018; West et al., 2020; Yang et al., 2020), early life stress (ELS) (Chaloner and Greenwood-Van Meerveld, 2013; Mohammadi et al., 2016; Pierce et al., 2016, 2018; Fuentes et al., 2017, 2021; Fuentes and Christianson, 2018; Ligon et al., 2018), and repeated variable stress (RVS) (Hammack et al., 2009; Merrill et al., 2013; Merrill and Vizzard, 2014; Hattori et al., 2019; Girard et al., 2020). As shown by the findings from these models, chronic stress could induce functional and histopathological changes at multiple levels of the micturition pathway, providing insight into the underlying mechanisms and potential treatments of LUTD.

However, different psychological stressors have their own natures and likely stimulate separate brain regions to elicit appropriate responses (Scharf and Schmidt, 2012). Due to this stressor-dependent effect, stress animal models have presented varied LUTD-related results, which in some cases are conflicting. Additionally, a stress model frequently mimics only limited aspects of LUTD in humans and is tailored to answer a specific experimental theory. Moreover, each stress animal model has its own advantages and disadvantages, which should be discussed in detail. Therefore, the aim of our study is to provide a state-of-the-art overview of stress-related animal models, hoping to obtain mechanistic insights, facilitate model choices and propose novel treatment strategies for LUTD.

## Methods

A comprehensive electronic literature search was conducted using the PubMed database to identify publications related to chronic psychological stress-induced LUTDs. The keywords included the following terms: “stress,” “animal model,” “bladder,” “lower urinary tract,” “pain,” “voiding,” “micturition,” “micturition frequency,” “urinary frequency,” or “frequency,” using single or combination of words. The search was restricted to studies published between January 2000 and August 2021. Each article’s title and abstract were reviewed for their appropriateness and relevance to the topic. The reference lists from the articles identified by this search strategy were also examined to find additional sources.

## Basic Assessment Methods for Chronic Stress-Related Animal Models

Lower urinary tract dysfunction, such as OAB or IC/BPS, is commonly a symptom-based diagnosis, and symptoms are associated with urine storage and voiding phases. The hallmark symptoms appear to be urinary frequency and bladder-related pain. Due to the subjective nature of symptoms, the animal cannot communicate intent or relate their urinary symptoms to researchers. Therefore, it is necessary to apply surrogate markers for the symptoms of LUTD instead of intentional acts. Several technical methods have been developed to quantify the voiding activities and bladder nociception on the basis of feasibility and physiological relevance (Lai et al., 2015b).

### Assessment of Voiding Activities

Three common methods have been applied to assess voiding activities, which include micturition cage, urine blotting patterns, and cystometry. In the approach of the micturition cage, a single animal will be placed into a micturition cage to record voiding frequency, interval, and volume, as well as water intake in real time (Wood et al., 2001). For the approach of urine blotting patterns, a blotting paper is laid on the bottom of the cage (below the mesh floor) to absorb the urine, and voiding activities are quantified as urine spots visualized under UV lamp or by methylene blue staining (Boudes et al., 2011). Cystometry allows the quantification of various urodynamic parameters during bladder infusion, such as voiding pressure, contraction duration, intercontraction intervals (ICIs), and non-voiding contractions. Cystometry serves as a standardized methodology and can be combined with visceromotor response (VMR) measurement.

### Assessment of Bladder Nociception in Animal Models

Two main methods have been developed to determine bladder nociception in animals. VMR, a pseudo-affective reflex, is considered to be a reliable and reproducible marker to quantify pain sensation and study analgesic agents (Ness et al., 2001; Pierce et al., 2016; Gao et al., 2017). It is recorded as electromyogram responses of the abdominal external oblique musculature to graded bladder distention in anesthetized animals. VMR threshold pressure is defined as the bladder pressure evoking VMR and used to assess the visceral pain sensitization. Other indicators are also used to evaluate VMR, including amplitude, duration, and area under the curve (AUC) (Ness and Elhefni, 2004; Gao et al., 2017). VMR could be inhibited by analgesics or augmented by the presence of stress or inflammation (Castroman and Ness, 2001).

Alternatively, von Frey suprapubic hyperalgesia is used to be a surrogate metric for assessing referred bladder hyperalgesia. It is measured by response to suprapubic mechanical stimuli with a calibrated series of nylon Von Frey monofilaments (Rudick et al., 2007; Lee et al., 2015).

## Animal Models of Chronic Stress-Induced Lower Urinary Tract Dysfunction

**FIGURE 1:**
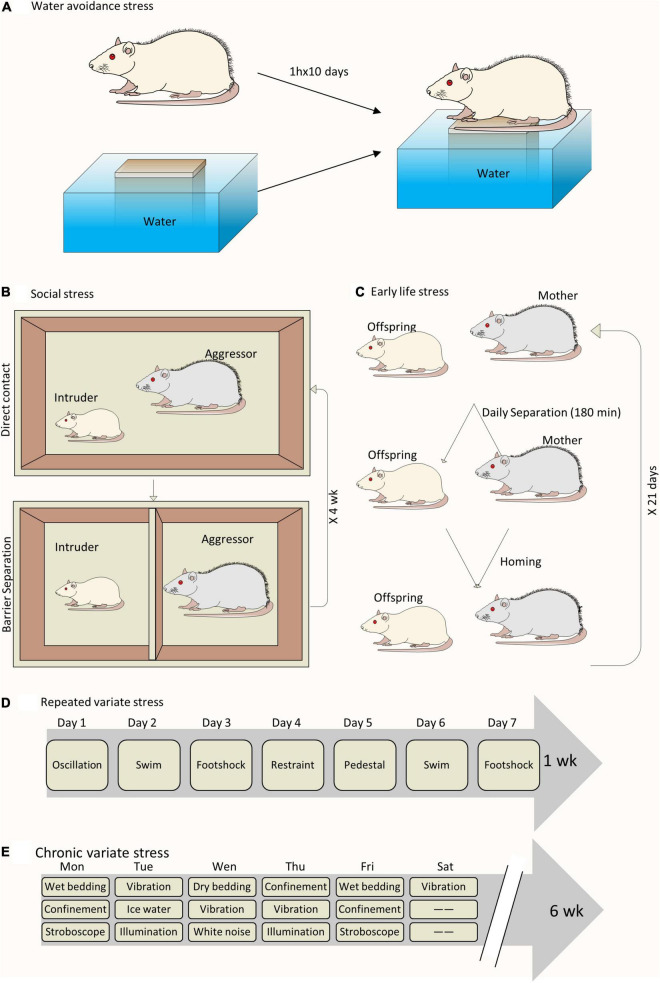
Schematic drawing of different animal models to study chronic psychological stress-induced bladder dysfunction. **(A)** A sample of WAS model. **(B)** A sample of social stress model. **(C)** A sample of ELS model. **(D)** A sample of RVS model. **(E)** A sample of chronic variable stress model.

A wide range of animal models have been developed to study the close link between chronic psychological stress and LUTD (Figure 1). It seems inconceivable to use a single animal model to replicate all the facets of LUTD in humans. It is better to carefully select certain species and stress paradigms before the experiments. The functional and histopathological changes are mainly decided by stressors *via* particular mechanisms (Table 1).

**TABLE 1:** Animal models of chronic psychological stress-induced bladder dysfunction.

Model	References	Species	Sex	Micturition frequency	Bladder hyperalgesia	Main advantage	Best translational research use
Water avoidance stress	Yamamoto et al., 2012; Bazi et al., 2013	Sprague-Dawley rats	Female or male	↑	-	Well and easily established, economic, objective, reproducible	Studying of psychological stress-induced voiding dysfunctions like interstitial cystitis or bladder pain syndrome or overactive bladder phenotype
	Robbins et al., 2007; Smith et al., 2011; Lee et al., 2015; Ackerman et al., 2016; Gao et al., 2017; Matos et al., 2017; Kullmann et al., 2019; Holschneider et al., 2020b; Sanford et al., 2020	Wistar-Kyoto rats	Female	↑	↑		
	Cetinel et al., 2005; Saglam et al., 2006; Zeybek et al., 2007; Okasha and Bayomy, 2010	Wistar albino rats	Female	-	-		
	Dias et al., 2019	Wistar rats	Female	↑	-		
	McGonagle et al., 2012	Swiss-Webster mice	Male	↓	-		
	West et al., 2021	C57BL/6J mice	Female	↑	-		
Social stress	Long et al., 2014; Weiss et al., 2015; Butler et al., 2018	Swiss-Webster mice	Male	↓	-	Ethological relevant, reproducible	Studying of psychological stress-induced voiding dysfunctions, particularly urinary retention
	Wood et al., 2009, 2012, 2013	Sprague-Dawley rat	Male	↓	-		
	Mingin et al., 2014; Yang et al., 2020	FVB mice	Male	↑ or ↓	-		
	Mann et al., 2015; Mingin et al., 2015; West et al., 2020	C57BL/6 mice	Male	↑ or ↓	-		
Early life stress	Pierce et al., 2016; Fuentes et al., 2017, 2021; Pierce et al., 2018	C57BL/6 mice	Female or male	↑	↑	Ethological relevant	Studying of adult bladder dysfunction after early psychological stress exposure
	Mohammadi et al., 2016; Ligon et al., 2018	Long-Evans rats	Female	↑	↑		
Repeated variable stress	Merrill et al., 2013; Merrill and Vizzard, 2014	Wistar rats	Male	↑	-	Reproducible, lack of habituation	Studying of different daily stress-induced voiding dysfunctions
	Hattori et al., 2019	Sprague-Dawley rats	Female	↑	-		
	Girard et al., 2020	Transgenic mice	Female	↑	↑		
Chronic variable stress	Yoon et al., 2010; Han et al., 2015	Sprague-Dawley rats	Female	↑	-	Lack of habituation	Study of bladder functional changes under long-term stressful condition
Intermittent restraint stress	Ihara et al., 2019	C57BL/6 mice	Male	↑	-	Easily established, economic, reproducible	Study of stress-induced nocturia

### Model of Water Avoidance Stress

It is common to all investigations on this WAS model to place a single rodent on a platform centered in the middle of a water-filled basin. The rodent will try to avoid the adverse stimulus (water) by staying on the platform, causing potent psychological stress. WAS has previously been shown to be associated with increased anxiety-like behavior in rodents and visceral hyperalgesia in their guts (Bradesi et al., 2005). Currently, it is also used to replicate chronic stress-induced LUTD in rodents. The WAS model is found to present high construct and face validity to bladder hypersensitive syndromes.

However, the choices of rodent species, gender, and stress paradigm vary in different studies. Selecting the rodent species apparently limits the selection of specific strains based on their own characteristics. Wistar-Kyoto (WKY) rats are genetically predisposed to elevated levels of anxiety (Pare, 1992), commonly regarded as a standard model animal for testing visceral nociception. Sprague-Dawley (SD) rats are generally considered to be a low or moderate anxiety strain, and their use for stress protocols seems controversial due to two different studies with opposite results (Robbins et al., 2007; Bazi et al., 2013). Mice are seldom used for WAS paradigms, despite a certain report of successful application (McGonagle et al., 2012). When referring to gender, female rodents are usually chosen on account of the greater illness severity of female voiding disorders in women (Stewart et al., 2003; Lifford and Curhan, 2009). A diverse duration exists for WAS model establishment by different research groups. Most investigators, including our group, have subjected rodents to WAS 1 h per day for 10 consecutive days (Robbins et al., 2007; Smith et al., 2011; Ackerman et al., 2016; Gao et al., 2017). However, the stress paradigm could be low to 5 (Cetinel et al., 2005) or 7 (Bazi et al., 2013) days if rodents are exposed to WAS for 2 h daily.

Functionally, WAS exposure in rats potently produces a phenotype characterized by increased anxiety-like behaviors, urinary frequency, and bladder hyperalgesia (Smith et al., 2011; Bazi et al., 2013; Lee et al., 2015; Ackerman et al., 2016). Using micturition cage analysis, we first reported that female WKY rats with WAS exposure developed nearly double the voiding frequency and nearly half the voiding interval as the controls (Smith et al., 2011). These voiding alterations persisted for approximately 1 month after WAS exposure. Similarly, Yamamoto et al. (2012) and Bazi et al. (2013) demonstrated increased voiding frequency and decreased ICI and volume in WAS SD rats compared to the controls. Additionally, a lower bladder pressure can trigger the voiding phase in WAS WKY rats, which suggests the appearance of WAS-induced bladder hypersensitivity (Gao et al., 2017; Wang et al., 2017). Most importantly, exposure of WKY rats to WAS produced sensitized and enhanced bladder nociceptive responses. They can manifest as early appearing and vigorous VMRs to graded (Robbins et al., 2007; Gao et al., 2017) or continuous (Lee et al., 2015; Gao et al., 2017) bladder distension, and also significant tactile hindpaw allodynia and suprapubic hyperalgesia to von Frey filament testing (Lee et al., 2015). Furthermore, once established, the increased tactile allodynia and bladder hyperalgesia could last over 1 month without continued stress exposure, possibly due to involvement of central augmentation and peripheral neuroplasticity (Lee et al., 2015).

Additionally, WAS could also result in different kinds of histopathological changes in rat bladder. Harvested bladder tissue from stressed rats showed inflammatory conditions, which include ulcerated areas, edema, vascular congestion, inflammatory cell infiltration, increased angiogenesis, and an increased number of mast cells in the mucosa (Cetinel et al., 2005; Saglam et al., 2006; Okasha and Bayomy, 2010; Smith et al., 2011; Bazi et al., 2013; Matos et al., 2017). Meanwhile, under electron microscopy, vacuole formation, dilated perinuclear cisternae, and dilatation in the intercellular spaces were also observed in the urothelium of WAS rats (Cetinel et al., 2005; Saglam et al., 2006; Okasha and Bayomy, 2010). Moreover, WAS exposure altered the mitochondrial function of urothelial cells and led to secondary impairment of urothelial function (Kullmann et al., 2019). Based on the above evidence, several investigators have tried to explore potential drugs to reverse these degenerative changes in stressed rat bladders. Cetinel et al. (2005) first reported the protective effect of melatonin on WAS-induced bladder degeneration in Wistar albino rats. After melatonin treatment, the impaired integrity of urothelial morphology and the increased number of mast cells in the mucosa were ameliorated in WAS rats. Saglam et al. (2006) noted that aqueous garlic extract protected the urothelium by preserving the urothelial mucous layer and reducing the number and degranulation of mast cells. Since then, several other drugs have also been tested to treat WAS-induced bladder degeneration in rats, which include taurine (Zeybek et al., 2007), quercetin (Okasha and Bayomy, 2010), epigallocatechin gallate (Bazi et al., 2013), cyclooxygenase-2 (COX-2) inhibitor (Yamamoto et al., 2012), silodosin (α-1 adrenoceptor antagonist) (Matos et al., 2017), and guanethidine (an adrenergic neuron blocking agent) (Kullmann et al., 2019). These studies have suggested certain possible and effective treatment options for chronic stress-related LUTD.

Based on these functional and histological findings, plenty of mechanisms were proposed to explain the pathophysiological basis of WAS-induced LUTD. Yamamoto et al. speculated that upregulated bladder COX-2 gene expression contributed to WAS-induced voiding frequency (Yamamoto et al., 2012). Dias et al. (2019). demonstrated significantly increased serum and urinary levels of nerve growth factor (NGF) in stressed rats, and blocking its high-affinity receptor tropomyosin-related kinase subtype A (Trk A) prevented WAS-induced voiding changes. NGF is a well-known molecule that contributes to the bladder afferent hypersensitization and hyperexcitability (Yoshimura et al., 2014). Specifically, the role of sensitized C-fiber afferents in bladder hypersensitivity was demonstrated in our previous study, in which WAS WKY rats showed a low threshold but enhanced VMR response to bladder cold saline infusion in comparison with the controls (Gao et al., 2017). Moreover, the exaggerated activity of the peripheral sympathetic nervous system may also contribute to bladder dysfunction and pain in WAS rats, possibly suppressed by the administration of silodosin (Matos et al., 2017) and guanethidine (Kullmann et al., 2019). Beyond these peripheral mechanisms, WAS-induced bladder hyperalgesia was correlated with increased spinal glutamate neurotransmission attributable to the downregulation of glial glutamate transporter 1 (Glt-1) receptors (Ackerman et al., 2016). Exogenous Glt1 upregulation *via* ceftriaxone treatment attenuated hyperalgesia and voiding dysfunction after WAS exposure (Ackerman et al., 2016; Holschneider et al., 2020a). Our brain mapping results showed greater activation in cerebral regions of the micturition circuit responsive to urgency, viscerosensory perception, and its relay to motor regions coordinating imminent bladder contraction, in particular the pontine micturition center (PMC), periaqueductal gray (PAG), thalamus, cingulate, and insula (Gao et al., 2017; Wang et al., 2017). Voluntary exercise or ceftriaxone administration could diminish bladder hypersensitivity and attenuate brain regions related to nociceptive and micturition circuits (Holschneider et al., 2020a,b; Sanford et al., 2020).

Of note, WAS exposure in mice results in different types of voiding dysfunction. In West’s study, female C57BL/6 mice were exposed to WAS for 1 h per day for 10 days, which produces an OAB phenotype with increased voiding frequency and enhanced bladder contractile responses (West et al., 2021). In contrast, McGonagle et al. (2012) applied the WAS protocol (1 h daily for 4 weeks) in male Swiss Webster mice and reported an altered voiding phenotype with a decreased voiding frequency and increased average voided volumes, seemingly attributable to the alteration of the calcineurin–nuclear factor of activated T-cells (NFAT) pathway in the bladder.

Accordingly, WAS is a less expensive but more objective means to study underlying mechanisms of chronic stress-induced LUTD and evaluate the impact of pharmacological agents. This WAS-induced voiding phenotype is much close to physiological condition without bladder irritants or directly inflicting bladder inflammation. The technique is easily done and outcomes are highly reproducible. One limitation of the WAS model seems to be restricted to certain rat strains such as anxiety-prone ones. Wistar albino rats have also been chosen in some WAS protocols, while none of them have reported bladder hyperalgesia after WAS exposure (Cetinel et al., 2005; Saglam et al., 2006; Zeybek et al., 2007). Altogether, these considerations can demonstrate that rodents—particularly the anxiety-prone ones—provide multiple accesses to effectively mimic the key features of human LUTD.

### Model of Social Stress

Social stress (or social defeat), usually mimicking bullying–childhood violence, has been shown to induce distinct voiding dysfunction in rodents. In the common social stress paradigm, the subordination of one male by another larger and more aggressive male could increase the risk of anxiety and depression (West et al., 2020). Male mice are the most frequently employed mice to explore this stress-related LUTD. This could be exemplified by Chang et al.’s study, in which a single submissive FVB mouse was repeatedly exposed (4 weeks) to an aggressive male C57BL/6 mouse for 1 h a day followed by 23 h of barrier separation (Chang et al., 2009). Several other investigators conducted similar paradigms to induce social stress (Long et al., 2014; Mingin et al., 2014, 2015; Weiss et al., 2015; Yang et al., 2020). Alternatively, the duration of social stress in the study Mann et al. (2015) was reduced to 2 weeks, and the direct daily exposure of male mice to aggressive ones was reduced to 10 min. In West’s study, pairs of male C57BL/6 mice were exposed to an aggressor for 1 h/day for 10 days (West et al., 2020). Instead of mice, Wood et al. (2009) exposed a male SD rat to a larger Long-Evans resident for a 30-min session with direct or indirect contact on 7 consecutive days.

After social stress exposure, rodents develop micturition alterations resembling partial bladder outlet obstruction in many ways. Chang et al. (2009). showed that socially stressed FVB mice had a lower voiding frequency, a greater voiding volume, and an increased threshold than matched controls, which suggests a social stress-induced urinary retention. Long et al. similarly determined that socially stressed C57BL/6 mice could present a single, large void pattern (Mann et al., 2015). Apart from mice, such social stress-related urinary LUTD was also noted in a rat model, and only stressed rats presented numerous non-micturition-related contractions (Wood et al., 2009). Several other studies also corroborated the above findings of stress-induced urinary LUTD (Wood et al., 2013; Long et al., 2014; Mingin et al., 2014).

Furthermore, social stress leads to hypertrophy and remodeling of the rodent bladder (Chang et al., 2009; Wood et al., 2009, 2012). Chang et al. (2009) demonstrated a 2-fold increase in the bladder-to-body mass index in socially stressed FVB mice. In this study, stressed bladders showed alterations in myosin heavy chain isoform expression and DNA synthesis mediating bladder wall reconstruction (Chang et al., 2009). Mann et al. (2015) also found that social stress induced a higher bladder-to-body mass index and thicker bladder wall. Moreover, the occurrence of bladder inflammation was evidenced by increased bladder NGF and histamine protein expression in socially stressed FVB mice (Mingin et al., 2014). In contrast, Mann et al. (2015) demonstrated an intact urothelial barrier and normal vascularity in socially stressed C57BL/6 bladders.

These aforementioned studies shed some light on potential mechanistic pathways linking social stress to LUTD from peripheral involvement to central participation. One of them involved a clear-cut change in the bladder calcineurin–NFAT pathway, the inhibition of which by cyclosporine A resulted in amelioration of the post-stressed abnormal voiding phenotype (Long et al., 2014; Weiss et al., 2015). A recent study showed that a social stress-altered voiding phenotype was associated with desensitized bladder sensory nerves by greater urothelial acetylcholine release and purinergic responses (West et al., 2020). Additionally, corticotropin-releasing factor (CRF), a stress-related neuropeptide, may be intrinsically involved in this type of LUTD. Social stress increased the number of CRF-immunoreactive neurons and the expression of CRF mRNA in Barrington’s nucleus (PMC), which possibly leads to subsequent bladder dysfunction (Wood et al., 2009). This CRF mRNA expression in Barrington’s nucleus remained elevated after 1 month of recovery from social stress, correlated with persistent voiding dysfunction (Butler et al., 2018). Administration of a CRF1 antagonist or shRNA targeting CRF in Barrington’s nucleus could improve social stress-induced retention (Wood et al., 2013). Another elevated neuropeptide brain-derived neurotrophic factor was also noted in rodent serum after social stress exposure, and a short-term subanesthetic dose of ketamine reduced its expression and reverse the trend of stress-related infrequent voiding (Yang et al., 2020).

In addition, the effect of social stress on the bladder seems context- and duration-dependent. After social stress, increased voiding frequency can occur first (Mingin et al., 2014, 2015) but voiding frequency decreases later with enhanced stress intensity (Mingin et al., 2014). Mingin et al. (2015) observed decreased ICI, voided volume, bladder capacity, and elevated transient receptor potential vanilloid 1 (TRPV1) expression in socially stressed C57BL/6 mice. Inhibiting TRPV1 was able to significantly restore bladder overactivity and reduce afferent bladder nerve activity in stressed mice (Mingin et al., 2015). Moreover, TRPV1 knockout mice also showed no social stress-induced bladder dysfunction (Tykocki et al., 2018). TRPV1 has emerged as a key regulator of bladder sensory processes and as a potential therapeutic target for LUTD (Vanneste et al., 2021).

In contrast to other stressors, social stress is ethologically relevant and suitable to create more innovative and effective management for children presenting voiding dysfunction with bullying or childhood violence history. Context- and duration-dependent effects are typical features of the social stress paradigm. However, an apparent limitation comes from its uneconomic process, such as essential single cage housing to avoid developing social rank hierarchies even among sibling littermates. In addition, another compelling issue is whether social stress can exert an effect on female rodents because current reports are all focused on males.

### Model of Early Life Stress

Early life stress, or early adverse events, has been shown to serve as a strong predictor of a painful voiding phenotype later in life. Several translational studies have tried to examine the impact of ELS on the bladder using two main types of animal models, namely neonatal maternal separation (NMS) (Pierce et al., 2016, 2018; Fuentes et al., 2017) and neonatal odor-shock conditioning (NOSC) (Mohammadi et al., 2016; Ligon et al., 2018). To produce NMS, mouse pups are removed from their dam for 3 h/day from postnatal days 1 to 21 (Pierce et al., 2016, 2018; Fuentes et al., 2017). NMS could also be combined with acute WAS as adults to imitate ELS with later adult trauma (Pierce et al., 2016; Fuentes et al., 2017). Pierce et al. (2016) provided evidence that NMS in female C57BL/6 mice was capable of inducing bladder hypersensitivity, manifested as a significantly increased VMR to bladder distension. Exposure to adult WAS further increased bladder sensitivity and micturition rate in these NMS mice, strongly suggestive of the necessity of additional stress for induction of painful LUTD phenotype (Pierce et al., 2016). Further molecular evidence from this study demonstrated neurogenic bladder inflammation and disruption of the proper hypothalamic–pituitary–adrenal (HPA) axis function after NMS. The HPA axis is well known to regulate stress response and affect the perception of pain (Habib et al., 2001). Pierce’s later study proved that voluntary exercise could attenuate NMS-induced behavioral outcomes in female C57BL/6 mice (Pierce et al., 2018), quite similar to the findings in WAS rats with voluntary exercise (Holschneider et al., 2020b; Sanford et al., 2020). Beyond female mice, male mice could also exhibit NMS-induced bladder hypersensitivity (Fuentes et al., 2017), urologic chronic pelvic pain, and mast cell degranulation in the bladder (Fuentes et al., 2021), all of which could also be ameliorated by voluntary exercise (Fuentes et al., 2021). Compared to mouse species, SD rat pups subjected to 6 h of daily NMS presented intermittent urinary retention with delayed withdrawal of the immature perigenital-bladder reflex (Wu and de Groat, 2006).

The NOSC model was initially developed to mimic attachment to an abusive caregiver. Mohammadi et al. (2016) utilized this model to condition male and female Long-Evans pups on postnatal days 8–12 to predictable odor-shock, unpredictable odor-shock, or odor-only treatment. In adulthood, female rats with unpredictable NOSC presented a significantly increased urine voiding volume and decreased detrusor contractile responses. Conversely, male rats showed no difference in voiding behavior or detrusor muscle activity after stress exposure, indicative of a female predominance of NOSC on urinary bladder function. Mohammadi’s recent study demonstrated that linaclotide, a guanylate cyclase-C agonist, could significantly reduce NOSC-induced bladder hypersensitivity (Ligon et al., 2018). In contrast to the NMS model, bladder inflammation was not found in this NOSC model.

The main advantage of the ELS model is to initiate adult physiological changes by a childhood psychological intervention, which expands our knowledge of how ELS predisposes an individual to develop LUTD during adulthood. This model has both face validity and construct validity, as well as incorporation of a non-invasive induction. Given that NMS dramatically affects the function of the HPA axis, it is important to control environmental stimuli during both the neonatal and adult periods.

### Model of Repeated Variable Stress

To mimic the variety of life stressors experienced by humans on a daily basis, an RVS model is established to elucidate the connection between stress and voiding dysfunction (Merrill et al., 2013; Merrill and Vizzard, 2014). Generally, rodents assigned to RVS are exposed to a 7-day stress protocol with a single stressor daily, which includes oscillation, swim, footshock, restraint, pedestal, swim, and footshock. After RVS exposure, both male Wistar rats (Merrill et al., 2013; Merrill and Vizzard, 2014) and female transgenic mice (Girard et al., 2020) exhibited increased voiding frequency and somatic sensitivity similar to IC/BPS. Elevated inflammatory markers (NGF, histamine, myeloperoxidase, and chemokines) have been found in stressed bladder tissue (Merrill et al., 2013; Girard et al., 2020), which is indicative of an RVS-induced inflammatory milieu of the bladder. Conversely, Hattori et al. (2019) examined the effect of RVS on female SD rats and found a significantly decreased micturition frequency and ICI in conscious and unconscious cystometry, respectively.

Several plausible mechanisms were offered for the RVS-induced voiding alterations. Merrill and Vizzard (2014) identified significantly increased TRPV4 expression in the urothelium of RVS rats and the intravesical TRPV4 antagonist HC067047 attenuated this RVS-induced bladder dysfunction. Notably, a more recent study by this research group reported a change in pituitary adenylate cyclase-activating polypeptide (PACAP) receptor signaling in lumbosacral dorsal root ganglions and spinal cord but not in the urinary bladder (Girard et al., 2020). PACAP, a type of neuropeptide, is expressed in neural and non-neural components of LUT and exhibits neuroplastic changes under pathological conditions that include psychological stress, inflammation, and neuron injury (Girard et al., 2017). Intravesical administration of a PACAP receptor antagonist could contribute to decreased voiding frequency and pelvic sensitivity following RVS exposure (Girard et al., 2020). Moreover, administration of the α1-adrenoceptor antagonist naftopidil orally could partly prolong ICI in female SD rats subjected to RVS (Hattori et al., 2019).

The main advantage of the RVS protocol is that it is well-established, reproducible, and more relevant to human daily life stressors. Also, novel stressor exposure on a daily basis could lead to a lack of habituation.

### Model of Chronic Variable Stress

Chronic variable stress (CVS), or the chronic mild stress model, was initially utilized in the psychological research field for studies on depression. Similar to RVS, CVS included a series of stressors. In Yoon’s protocol, female SD rats were exposed to scheduled stress environments that include starvation, low temperatures (4°C), immobilization, and changes in the diurnal rhythm for 7, 14, and 28 days (Yoon et al., 2010). In this study, voiding frequency in stressed rats significantly increased with the prolonged duration of stress exposure, possibly related to higher bladder contractility caused by higher expression of Rho-kinase (one of the major mediators for muscle contraction and relaxation responses) in the bladder tissue. CVS also induced increased expression of types I and III collagen in bladder tissue, which suggests bladder fibrosis for bladder stability–overactivity (Yoon et al., 2010). Additionally, Han et al. (2015) applied a modified CVS protocol with a series of stressors for 6 weeks, such as confinement in a small cage, white noise during the dark phase, stroboscope, light on at night, intruder sound, and food and water deprivation. In Han’s study, stressed female SD rats also showed bladder hypersensitivity in cystometry such as increased micturition frequency and decreased micturition volume and ICI. Consistent with Yoon’s study above, Han’s further evaluation demonstrated increased RhoA/Rho-kinase expression and decreased neuronal nitric oxide synthase expression in the stressed bladder wall, which indicates a hyperactive bladder with a lower relaxation capacity (Han et al., 2015).

The pivotal feature of the CVS model is to utilize various unpredictable stressors to investigate the micturition pattern alterations reflecting long-term stressful human life events. Different stressors may contribute to the absence of adaptation. While obviously, establishing the CVS model needs much more time and total experimental cost.

### Intermittent Restraint Stress

Intermittent restraint stress (IRS), a notable psychological stressor to rodents, is also applied to study stress-induced LUTD. In Ihara’s study, male C57BL/6 mice were subjected to IRS during zeitgeber times 4–6 for 2 h/day for 5 consecutive days and exhibited higher voiding frequency and smaller urine volume or voiding in the light (sleep) phase (Ihara et al., 2019). This voiding phenotype such as nocturia may be pertinent to changes in circadian bladder function due to the dysregulation of clock genes such as Per2. Amending the circadian rhythm by Per2 inhibition could reduce voiding frequency and increase bladder capacity during the light phase in IRS mice. Mann’s reports, however, demonstrated that water-restraint stress (9 a.m. to 1 p.m., 4 h/day for 21 days), another type of restraint stress, could not produce functional or histopathological changes in the bladder of the male C57BL/6 mice (Mann et al., 2015). These differences between the findings of Ihara and Mann were possibly related to the differences in restraint stress type, stress duration, and daily stress schedule. The IRS model seems relatively cost-effective and straightforward to implement, but it carries some caveats including stress habituation or a lack of ecological relevance (Koolhaas et al., 2006). The voiding phenotypes may be influenced by different IRS protocols.

### Others

Other stressors such as intraspecies emotional communication (IEC) and foot shock stress (FSS) are also applied to explore the mechanisms underlying stress-induced LUTDs. IEC produced purely psychological stress without physical stress intervention (Gomita et al., 1989). Male WKY rats with IEC 2 h/day for 7 days developed greater micturition frequency and voided volume per day, which probably results from IEC reinforced muscarinic receptor 3-mediated contractions *via* CRF1 pathway (Seki et al., 2019). FSS was a readily controlled physical stressor to produce behavioral and neurochemical changes (Kant et al., 1983; Rivier and Vale, 1987; Henry et al., 2006). FSS-stressed female SD rats displayed bladder hypersensitivity, which could be significantly alleviated by blocking spinal CRF2 (Robbins and Ness, 2008) or oxytocin systematic treatment (Black et al., 2009). Acute stressors such as acute immobilization (Boucher et al., 2002, 2010) and acute FSS (Robbins et al., 2011; DeBerry et al., 2015; Ness et al., 2018) can also induce LUTDs, which are not discussed detailly in this study.

## Sex- and Strain-Specific Differences in Animal Models

Various epidemiological studies have demonstrated that gender bias in human LUTD is apparent and that women are more vulnerable to the development of LUTD (Stewart et al., 2003; Lifford and Curhan, 2009). Therefore, as described above, female rodents are usually employed to study stress-related LUTDs. However, one important point should be taken into account: the animal’s sex and hormones may contribute to the alterations in micturition following chronic stress exposure. For instance, male or female mice with WAS exposure showed significant differences in voiding frequency (McGonagle et al., 2012; West et al., 2021). Similar results can be found in the male or female rats in response to NOSC exposure (Mohammadi et al., 2016). Several hypotheses attempt to explain the sexually dimorphic effects of chronic stress on micturition. One hypothesis is the involvement of estrous influences. Estrogen is well known to be effective on female bladder contractility and stability in both humans and animals (see review Hanna-Mitchell et al., 2016). Moreover, estrogen could also be affected by chronic stress. For example, female SD rats with CVS exposure presented significant increases in voiding frequency and bladder contraction, together with a significant decrease in estrogen. Another hypothesis is the sex-specific effects of stress exposure on the HPA axis. As shown by Sterrenburg’s study, CVS could induce sex-specific differences in methylation and expression of the CRF gene in animal brains (Sterrenburg et al., 2011).

Another point that should be noted is that the results of lower urinary function tests vary among strains. As mentioned above, WKY rats are more genetically predisposed to anxiety than SD rats. Robbins’s study reported that chronic psychological stress (WAS) significantly enhanced bladder nociceptive responses only in WKY rats rather than SD rats (Robbins et al., 2007). Similarly, WAS exposure induced different voiding results obtained from two mouse strains (C57BL/6 and Swiss Webster) (McGonagle et al., 2012; West et al., 2021). These differences in urinary function tests may be related to strain-specific bladder physiology or behavioral factors. Other non-stress-related studies have been also demonstrated the strain-specific effect on urinary function (Yu et al., 2014; Bjorling et al., 2015).

However, more studies are necessary to determine the sex- and strain-specific differences in stress-related LUTD models.

## Implications for Integrated Mechanisms

**FIGURE 2:**
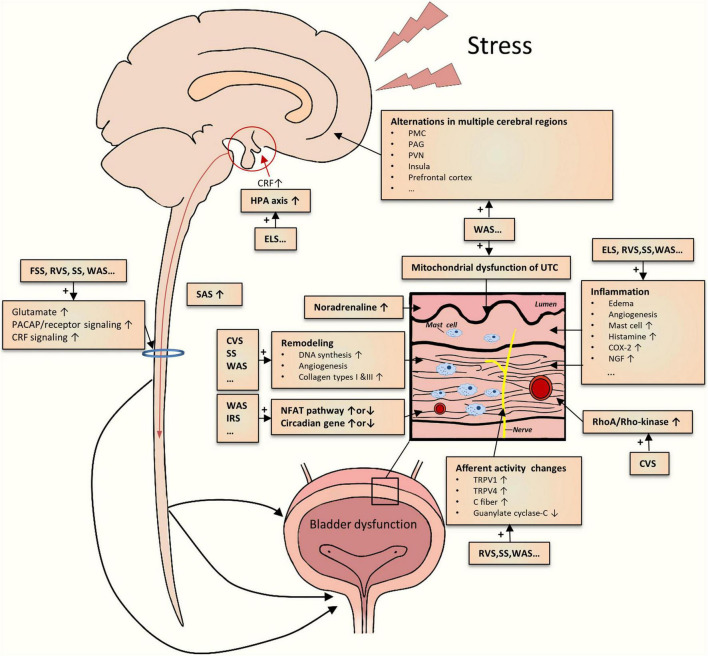
Schematic drawing of the summarization of possible mechanisms underlying the chronic psychological stress-induced LUTD. The current studies demonstrate multiple central and peripheral alterations contributing to bladder dysfunction.

As mentioned above, various studies on different chronic psychological stress models have yielded incremental pieces of evidence for stress-induced LUTDs. However, the results from them appeared to be disparate for a definite conclusion. By integrating the evidence together, we could create a reasonable picture of the pathophysiology of stress-induced LUTDs (Figure 2). The possible mechanisms are roughly divided into two main classes as follows.

### Central Stress Mechanisms

Limbic–hypothalamic–pituitary–adrenal axis is crucial to coordinate behavioral, physiological, and molecular responses to chronic psychological stressors (Pacák, 2000; Ulrich-Lai and Herman, 2009; Ullmann et al., 2020). In particular, the paraventricular nucleus (PVN) of the hypothalamus is generally recognized as the main center regulating responses to stress (Pacák, 2000; Herman et al., 2008). PVN could secrete CRF, project into different sites (hypothalamus, brainstem, etc.), and stimulate adrenocorticotropic hormone secretion from the pituitary (Kalantaridou et al., 2010). CRF could mediate general responses to stress and other responses such as visceral hyperalgesia (Elman and Borsook, 2016; Chess-Williams et al., 2021; Huang et al., 2021). Other stress response-relevant brain regions include the prefrontal cortex, amygdala, and hippocampus (Kalantaridou et al., 2010). The disruption of the HPA axis has been reported in ELS rodents (Pierce et al., 2016). The functional alterations in these mentioned brain regions, together with other key areas (cingulate, insula, thalamus, etc.), were identified in WAS rats by brain mapping analysis (Wang et al., 2017; Holschneider et al., 2020a; Sanford et al., 2020) and in patients with LUTD by fMRI studies (Kleinhans et al., 2016). Additionally, aberrant expression and function of CRF in PMC were also noted in rodents subjected to WAS or social stress (Wood et al., 2009; Wang et al., 2017). Recently, Shimizu et al. (2021) postulated several brain molecules related to the stress-induced LUTDs, which include bombesin, angiotensin II, nicotinic acetylcholine receptor, nitric oxide, and H_2_S. Hence, psychological stress could significantly affect the cerebral regions related to stress responses and micturition circuit, which contributes to the development of LUTDs.

The sympathetic-adrenal system (SAS), another classic pathway for initiation and coordination of stress responses, appears to be involved in stress-induced LUTDs. SAS mainly consists of central (locus coeruleus and noradrenaline system) and peripheral (sympathetic nervous system and adrenal medulla) elements (Nargund, 2015). Activated by CRF *via* different approaches (Harris, 2015; Tache et al., 2018), SAS participates in multiple processes including micturition, mechanical hyperalgesia maintenance, and induction of viscrosensory hypersensitivity (Charrua et al., 2015). Activation of the SAS was partly proved by the results from the WAS and RVS models. Blocking SAS by the administration silodosin (Matos et al., 2017), guanethidine (Kullmann et al., 2019), or naftopidil (Hattori et al., 2019) could mitigate urinary dysfunctions after chronic stress exposure. These preclinical results were in line with the clinical studies, which confirmed the existence of SAS activation (Charrua et al., 2015).

Apart from these supraspinal mechanisms, spinal ones may also be engaged in stress-induced LUTDs. Chronic stress seems to enhance spinal signaling transductions, as evidenced by increased spinal glutamate neurotransmission (Ackerman et al., 2016; Holschneider et al., 2020a), PACAP (Girard et al., 2020), and CRF2 (Robbins and Ness, 2008) signaling transduction. Dorsal root ganglions may positively participate in these enhanced signal transductions (Girard et al., 2020). Spinal sensitization has been identified in other non-stressed animal models (Zvarova and Vizzard, 2006; Arms et al., 2013; Chen et al., 2016; Girard et al., 2016; Ozaki et al., 2018).

### Local Bladder Stress Mechanism

One theory for stress-induced LUTDs is the occurrence of bladder afferent hyperexcitability after chronic stress exposure. As mentioned above, stressed rodents commonly exhibited greater voiding frequency but lower pressure threshold and VMR threshold, which suggests bladder hypersensitivity and hyperalgesia. The activation of bladder C-fiber afferents (Gao et al., 2017) or TRPV1-sensitive bladder afferents (Mingin et al., 2015) may be involved in the stress-induced afferent hyperexcitability. Other candidates, such as TRPV4 pathway (Merrill and Vizzard, 2014), guanylate cyclase-C pathway (Ligon et al., 2018), and various inflammatory pathways (COX-2,NGF, histamine, myeloperoxidase, chemokines, etc.) (Yamamoto et al., 2012; Merrill et al., 2013; Mingin et al., 2014; Dias et al., 2019; Girard et al., 2020), may also be responsible for bladder afferent hyperexcitability.

In addition, chronic stress caused other functional and structural changes in the bladder tissues, particularly detrusor muscle and urothelium. Both *in vivo* and *in vitro* methods have demonstrated an enhanced detrusor contraction in stressed rodents (Yoon et al., 2010; Seki et al., 2019; West et al., 2021), partly related to bladder remodeling (Chang et al., 2009; Wood et al., 2009, 2012). The alteration in the urothelium, such as abnormal mitochondrial function, may contribute to increased bladder signal input (Kullmann et al., 2019). Particularly, chronic stress can bring about increased bladder inflammation that includes edema, inflammatory cell infiltration, and angiogenesis (Chess-Williams et al., 2021).

## Conclusion

Chronic psychological stress can impact LUT function with or without pathological consequences. This study provided a comprehensive overview of stress-related animal models for the study of LUTD and summarized the compensatory mechanisms underlying the emergence of LUTD. Variations in stress duration, the nature of chronic stressors, and differences in animal species, strains, and sex may contribute to different effects on the bladder. Based on the findings from these models, a variety of possible mechanisms and potential treatments have been demonstrated. Future studies should provide experimental means to improve the scientific basis for this common clinical problem, with the hope of ultimately expanding the therapeutic options for those patients with functional bladder disorders.

## Author Contributions

YG: data collection, analysis, and manuscript preparation. LR: project design and manuscript review. Both authors contributed to the article and approved the submitted version.

## Conflict of Interest

The authors declare that the research was conducted in the absence of any commercial or financial relationships that could be construed as a potential conflict of interest.

## Publisher’s Note

All claims expressed in this article are solely those of the authors and do not necessarily represent those of their affiliated organizations, or those of the publisher, the editors and the reviewers. Any product that may be evaluated in this article, or claim that may be made by its manufacturer, is not guaranteed or endorsed by the publisher.

## Abbreviations

COX-2: cyclooxygenase-2
CRF: corticotropin-releasing factor
CVS: chronic variable stress
ELS: early life stress
FSS: foot shock stress
HPA: hypothalamic pituitary adrenocortical axis
NFAT: nuclear factor of activated T-cells
NGF: nerve growth factor
PACAP: pituitary adenylate cyclase-activating polypeptide
PMC: pontine micturition center
PVN: paraventricular nucleus
RVS: repeated variable stress
SAS: sympathetic-adrenal system
SS: social stress
TRPV1: transient receptor potential vanilloid type 1
TRPV4: transient receptor potential vanilloid type 4
WAS: water avoidance stress.
